# Survival and HLA-B*57 in HIV/HCV Co-Infected Patients on Highly Active Antiretroviral Therapy (HAART)

**DOI:** 10.1371/journal.pone.0134158

**Published:** 2015-08-04

**Authors:** Leona Dold, Golo Ahlenstiel, Eva Althausen, Carolin Luda, Carolynne Schwarze-Zander, Christoph Boesecke, Jan-Christian Wasmuth, Jürgen Kurt Rockstroh, Ulrich Spengler

**Affiliations:** 1 Department of Internal Medicine 1, Rheinische Friedrich-Wilhelms University Bonn, Bonn, Germany; 2 Westmead Clinical School, University of Sydney, Sydney, Australia; 3 German Centre of Infection Research (DZIF), partner site Cologne-Bonn, Germany; University of Cincinnati College of Medicine, UNITED STATES

## Abstract

**Background and aims:**

HLA class I alleles, in particular HLA-B*57, constitute the most consistent host factor determining outcomes in untreated HCV- and HIV-infection. In this prospective cohort study, we analysed the impact of HLA class I alleles on all-cause mortality in patients with HIV-, HCV- and HIV/HCV- co-infection receiving HAART.

**Methods:**

In 2003 HLA-A and B alleles were determined and patients were prospectively followed in 3-month intervals until 2013 or death. HLA-A and B alleles were determined by strand-specific oligonucleotide hybridisation and PCR in 468 Caucasian patients with HCV- (n=120), HIV- (n=186) and HIV/HCV-infection (n=162). All patients with HIV-infection were on HAART. In each patient group, HLA class I-associated survival was analysed by Kaplan-Meier method and Cox regression analysis.

**Results:**

At recruitment the proportion of patients carrying a HLA-B*57 allele differed between HIV- (12.9%) and HCV-infection (4.2%). Kaplan Meier analysis revealed significantly increased mortality in HLA-B*57-positive patients with HIV-infection (p=0.032) and HIV/HCV-co-infection (p=0.004), which was apparently linked to non-viral infections. Cox logistic regression analysis confirmed HLA-B*57 (p=0.001), serum gamma-glutamyltranspeptidase (p=0.003), serum bilirubin (p=0.022) and CD4 counts (p=0.041) as independent predictors of death in HIV-infected patients.

**Conclusion:**

Differences in the prevalence of HLA-B*57 at study entry between HIV- and HCV- infected patients may reflect immune selection in the absence of antiviral therapy. When patients were treated with HAART, however, HLA-B*57 was associated with increased mortality and risk to die from bacterial infections and sepsis, suggesting an ambiguous role of HLA-B*57 for survival in HIV/HCV infection depending on the circumstances.

## Introduction

The course of untreated human immunodeficiency virus (HIV) infection is highly variable reflecting complex interactions between the virus, the host, and the environment. Although this variation is attributed to multiple factors, several studies identified human leucocyte antigen (HLA) class I loci as consistent host factors affecting clinical outcomes in HIV infection [1].

Importantly, HLA-B*57 was associated with delayed progression to AIDS and low-level viremia, while susceptibility to HIV infection was not affected. The association of HLA-B*57 with control of HIV is remarkably consistent among cohorts, even when accounting for differential HLA subtypes (HLA-B*5701 in Europe and the US, HLA-B*5703 in Africa) and diverse HI-viruses [2].

HLA class I molecules bind viral peptides to present them to the T cell receptors of CD8+ cytotoxic T lymphocytes. Thus, HLA-B*57-associated protection has been attributed to strong and broad cytotoxic T cell responses to immunodominant HLA-B*57 restricted epitopes on HIV proteins [3–5]. Alternatively, the association between infection outcomes and HLA alleles may reflect exceptional efficacy of HLA-B*57 to present epitopes to T cells, as has been reported for HLA-B*57 restricted epitopes on HIV gag and other HIV proteins [6–8]. Taken together, HLA-B*57-restricted cytotoxic T cell responses seem to effectively control HIV infection.

Hepatitis C virus (HCV) and HIV share the same routes of transmission, occurring frequently in the same groups, e.g. hemophiliacs, patients with intravenous drug abuse (IVDU), and HIV-positive men who have unprotected sex with men (MSM) [9]. HIV/HCV co-infection results in an accelerated course of chronic hepatitis towards cirrhosis and hepatocellular carcinoma (HCC).

Several studies indicated that control of HCV infection was also linked to HLA-B*57 [10–12]. Although the precise cause for this coincidence remains unclear, enhanced HLA-B*57 restricted cellular immunity might be involved. Finally, linkage disequilibrium between HLA-B*57 and neighboring genes such as *HCP5* and *ZNDR1* must be also considered [13].

Since it is unclear whether the protective effects of HLA-B*57 in HIV and HCV infection hold also true when HIV infection is treated by highly active antiretroviral therapy (HAART), we analyzed HLA class I associated survival in Caucasian HIV- and HCV-infected patients of the Bonn cohort.

## Patients and Methods

This study is based on the Bonn HIV and HCV cohort, which has already been reported in detail during transition to highly active antiretroviral therapy [14]. In 2002, the fraction of patients who achieved HAART-mediated control over HIV infection approached a steady state. Thus, we initiated a cross-sectional analysis of HLA-A and HLA-B alleles in the Bonn cohort and stratified patients into those who had HIV-1 infection, chronic hepatitis C and both, respectively [7, 14]. To avoid bias in the distribution of HLA alleles due to unbalanced contributions from non-Caucasian migrants between our study groups, we excluded non-Caucasian patients. 109 unselected healthy blood donors (75 males, 34 females; age 35 ± 12 years) served as a reference for the distribution of HLA alleles in the Caucasian background population. Patients were monitored every 3 months and any complications were recorded and classified according to the European modification of the Centers for Disease Control and Prevention criteria. In each disease group, we analyzed long-term all-cause-mortality and searched for associations between survival and distinct HLA alleles with a prevalence >5%. Finally, we identified independent predictors of mortality by stepwise Cox’s proportional-hazards analysis. The Bonn University ethics committee had approved the study. Written informed consent was obtained prior to entering a patient into the study.

In HIV-infected patients, antiretroviral therapy was given and adapted following the guidelines recommended by the International AIDS Society panels. In patients with chronic hepatitis C, we offered pegylated-interferon/ribavirin combination therapy unless contraindications were present.

Blood counts, liver-function tests (alanine and aspartate aminotransferases, alkaline phosphatase, bilirubin, and gamma-glutamyltranspeptidase), and CD4 counts were determined by routine procedures every 3 months.

HCV RNA testing was performed annually in serum, using a highly sensitive transcription-mediated amplification assay (Versant Qualitative HCV Assay/TMA; Bayer Diagnostics, Leverkusen, Germany; detection limit 5.3 IU/ml). HCV genotypes were determined by the Innolipa II line probe assay (Innogenetics, Zwijndrecht, Belgium). All patients were tested for HBsAg, anti-HBs, and anti-HBc with commercial assays (Abbott, Wiesbaden, Germany).

Initial HIV serology was based on a commercially available HIV antibody ELISA and p24 antigen assay (Abbott, Wiesbaden; Coulter, Hamburg, Germany). HIV loads were determined quantitatively using the NucliSens HIV-QT assay (Organon Teknika, Boxtel, the Netherlands). Amplified patient RNA was quantified with different electrochemiluminescent probes in the NASBA QR system (Organon Teknika, Boxtel, the Netherlands, detection limit 80 copies/mL).

After 2007, HIV and HCV viral loads were quantified by real time polymerase chain reaction (detection limits HCV 15 U/ml; HIV 50 copies/ml).

### Determination of HLA class I genotypes

HLA-A and HLA-B genotyping was performed using the INNO-LiPA line probe assays (Innogenetics), which are based on the reverse hybridization principle and enable to determine HLA class I alleles at intermediate resolution. To resolve ambiguous results, we used strand-specific PCR (One Lambda, Canoga Park, CA). Typing results were recorded with the 2-digit code to ensure a uniform level of HLA-resolution for all alleles (S1 File).

### Statistical analysis

Patients were stratified into the three groups: HIV, HCV or HIV/HCV infection. Differences in demographic, viral and biochemical parameters were compared between the patient groups by Student’s *t* test, chi-2 test, or the non-parametric Mann-Whitney test, as appropriate. The distribution of all HLA class alleles I with an allele frequency > 5% was compared between the groups at study entry using contingency tables and the multi-variate Kruskal-Wallis test. Multiple testing was corrected via the False Discovery Rate (FDR) according to Benjamini-Hochberg [15].

In each study group, we analyzed HLA associated survival using the Kaplan-Meier method and the Mantel-Cox log-rank test. Survival was assessed from study entry until death. All surviving patients were censored on 30.06.2013. Criteria for causes of death were checked independently by one of the investigators (JKR), who was unaware of the patients’ HLA allocation. Patients undergoing liver transplantation were statistically considered to represent analogues of liver-related death with the date of transplantation as “date of death”.

Survival was also studied by Cox’s proportional hazards analysis to identify factors independently associated with long-term mortality. First, each patient group was analyzed separately. Potential confounders included clinical features (sex, age, presence of hepatitis B, class of antiretroviral drugs, anti-HCV therapy and its outcomes), laboratory results (HCV and HIV loads, alanine aminotransferase, aspartate aminotransferase gamma-glutamyltranspeptidase, bilirubin, alkaline phosphatase, CD4 T cell counts), and distinct HLA class I alleles. Variables with a significant difference in univariate analysis (ANOVA) were entered stepwise into a forward conditional proportional-hazard regression model. Criteria for inclusion and exclusion of variables in the final model were p<0.05 and p>0.10, respectively. We also calculated the same type of regression model for all HIV-infected patients using “HCV infection” as an additional covariate.

All statistical analyses were done with the SPSS software package (version 22).

## Results

### Patient characteristics

**Fig 1:**
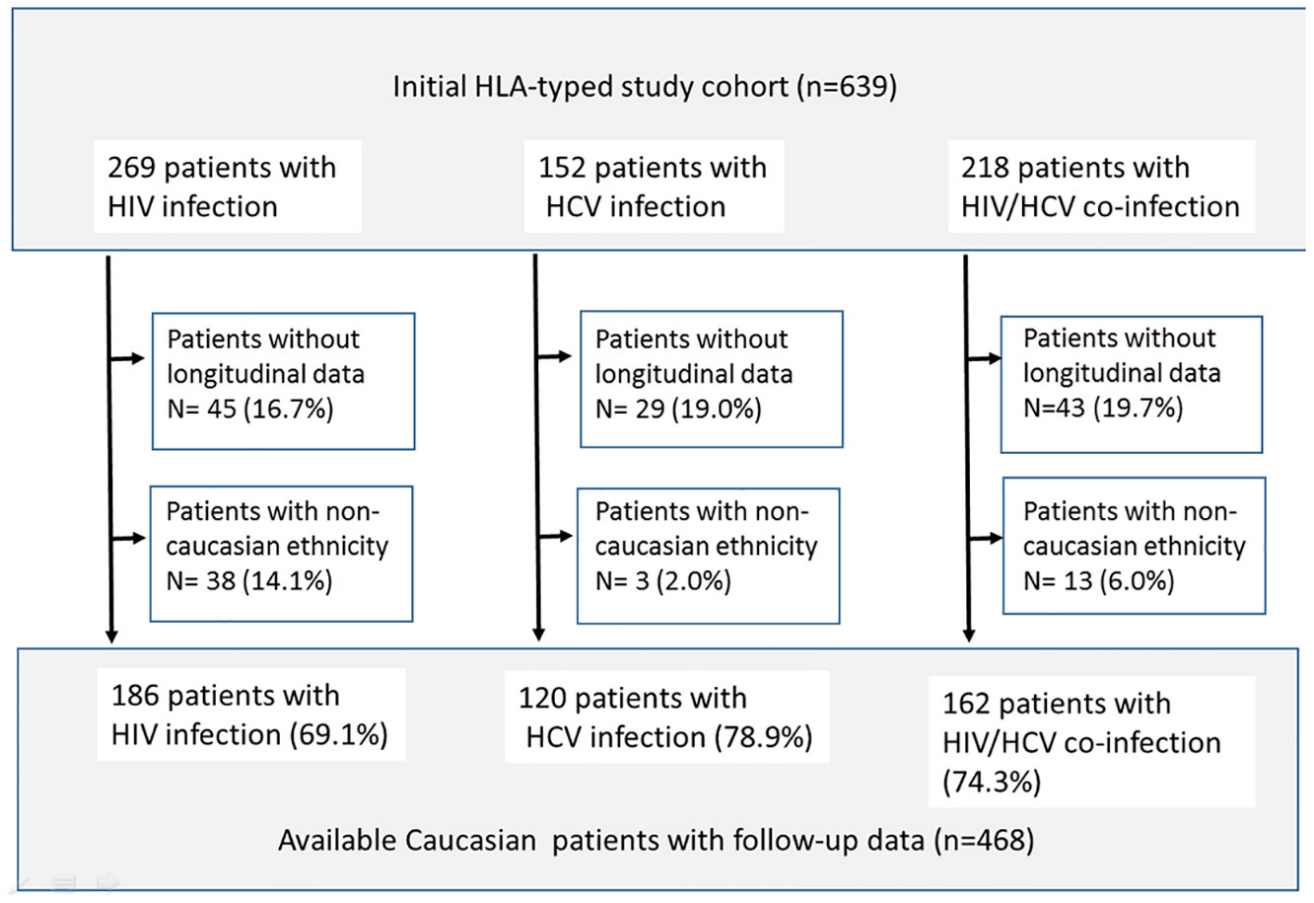
Long-term follow up and patient disposition in the Bonn HIV and HCV cohort.

The initial cohort comprised 639 HLA-typed patients who were stratified into the 3 study groups as summarized in Fig 1. Longitudinal follow-up data could not be obtained in 15.0–18.8 percent of patients in the study groups. Patients who did not have Caucasian descent (2% to 14.1% in each group) had to be excluded because their distribution of HLA alleles did not match the background population. To minimize recruitment bias, we excluded further 11 patients whose follow-up was less than 1 week. The remaining 468 patients (HLA-A n = 414, HLA-B n = 468) constitute the available study population, comprising 186 patients with HIV infection, 120 patients with chronic hepatitis C, and 162 patients with HIV/HCV co-infection. Demographic and clinical data are summarized in Table 1.

**Table 1:** Patient Characteristics in the Study Population.

	HIV	HCV	HIV/HCV
**Total number of patients (%)**	186 (39.7)	120 (25.7)	162 (34.6)
**Demographic data**			
Age (years) median (range)	42.7 (20.1–76.3)	44.2 (17.9–81.2)	41.0 (22.4–65.1)
Gender: male/female (% male)	162/24 (87.1)	103/17 (85.8)	144/18 (88.9)
**Risk category** n (%)			
Haemophilia	4 (2.1)	70 (57.4)	95 (58.6)
Intravenous drug abuse	3 (1.6)	12 (10.0)	41 (25.3)
Homosexual	99 (53.3)	0 (0)	10 (6.3)
Hterosexuell	22 (11.8)	0 (0)	5 (3.0)
Unknown routes of transmission	58 (31.2)	38 (31.7)	11 (6.8)
**HCV genotypes** n (%)			
1a		36 (30.0)	62 (38.3)
1b		41 (34.2)	29 (17.9)
2		12 (10.0)	13 (8.0)
3		13 (10.8)	35 (21.6)
4		7 (5.8)	5 (3.1)
Other genotypes or not done		11 (9.2)	18 (11.1)
**HCV viral loads (IU/ml)** ^#^	---------	1.747x10^6^ (1.570–2.780)x10^6^	1.849x10^6^ (1.580–3.450) x10^6^
**HCV therapy** n (%)	---------	73 (60.8)	66 (40.7)
**Hepatitis B serology** HBs AG positive n (%)	19 (10.2)	4 (3.3)	31 (19.1)
**HAART regimes** n (%)			
NRTI	16 (8.6) ^a)^		19 (11.7) ^e)^
NNRTI	27 (14.5) ^b)^		20 (12.4) ^f)^
PI	121 (65.1) ^c)^		96 (59.3) ^g)^
Other antiretrovirals	19 (10.2) ^d)^		20 (12.3) ^h)^
No antiretroviral drugs	3 (1.6)		7 (4.3)
**CD4 T-cell counts at study entry** ^#^(cells/μl)	444 (419–501)	-----------------	397 (377–469)
**CD4 T-cell counts at last observation** ^#^(cells/μl)	526 (96–582)	----------------	411 (380–505) ^A^
**AST (U/L)** ^#^	26 (24–34)	34 (32–47)	53 (48–75) ^B^
**ALT (U/L)** ^#^	34 (32–38) ^C^	56 (51–102)	64 (61–87)
**GGT (U/L)** ^#^	53 (51–75)	55 (49–102)	81 (77–121) ^D^
**Bilirubin (mg/dl)** ^#^	0.62 (0.59–0.82)	0.62 (0.54–0.77)	0.82 (0.74–1.16) ^E^
**HIV loads** (copies/ml)			
at study entry ^§^	0 (22.047)	-------	169 (13.595)
at last observation ^§^	0 (0)	-------	0 (23)

^#^ mean (95% confidence interval)

^§^ data are reported as median and interquartile range to account for deviation from the normal distribution

^A)^ p = 0.010 (HIV/HCV versus HIV)

^B)^ p<0.001 (HIV/HCV versus HIV) and p = 0.004 (HIV/HCV versus HCV)

^C)^ p<0.001 (both HIV versus HCV and HIV/HCV)

^D)^ p = 0.033 (HIV/HCV versus HIV)

^E)^ p = 0.052 (HIV/HCV versus HIV)

^*a)*^ 41.9% azidothymidine, 34.9% abacavir, 12.9% didanosine, 28.0% tenofovir, 75.8% lamivudine, 49.5% stavudine

^*b)*^ 23.7% efavirenz, 11.8% nevirapin

^*c)*^ 15.6% saquinavir, 33.8% lopinavir, 14.5% indinavir, 4.8% atazanavir, 0.5% darunavir, 2.7% fosamprenavir, 14.5% nelfinavir, 1.2% tipranavir 20.9% ritonavir (booster dose)

^*d)*^ 2.2% raltegravir, 1.1% enfuvirtide

^e)^ 40.1% azidothymidine, 19.1% abacavir, 12.3% didanosine, 37.7% tenofovir, 60.5% lamivudine, 29.6% stavudine

^*f)*^ 16.7% efavirenz, 6.2% nevirapin

^*g)*^ 9.8% saquinavir, 30.9% lopinavir, 11.1% indinavir, 6.2% atazanavir, 3.7% darunavir, 1.8% fosamprenavir, 9.9% nelfinavir, 18.5% ritonavir (booster dose)

^h)^ 1.2% raltegravir, 0.6% maraviroc

This table indicates that our groups represented typical patient populations for each type of infection. For instance, hemophilia was the major risk factor for patients with hepatitis C and HIV/HCV co-infection, while HIV mono-infected patients were predominantly MSM (Table 1). Liver enzymes were significantly higher in the two groups with hepatitis C and were highest in patients with HIV/HCV co-infection. HIV viral loads and CD4 counts indicated that HAART efficiently prevented HIV disease progression in almost all patients, but CD4 counts at last follow-up were significantly lower in HIV/HCV co-infection than in patients with HIV infection alone (p = 0.010) indicating impaired recovery of the immune system in HIV/HCV co-infection. HIV viral loads below the level of detection were achieved in 145 (78.0%) and 188 (72.8%) of the HIV-infected and HIV/HCV co-infected patients, respectively.

Fib-4 scores were significantly higher in HIV/HCV co-infected patients (2.48; 95% confidence interval: 2.94–2.93) than in patients with either HIV (1.31, 95% CI 0.98–1.65; p<0.001) or HCV (1.71, 95% CI 1.34–2.08; p = 0.028) mono-infection, reflecting enhanced liver damage in HIV/HCV co-infected patients. Seventy-three patients with chronic hepatitis C (60.8%) underwent combination therapy with pegylated interferon and ribavirin, and 34 treated patients (46.6%) achieved a sustained virological response. In HIV/HCV co-infection, a significantly lower percentage of patients (n = 66, 40.7%, p = 0.002) received anti-HCV combination therapy, which was successful in 25 (37.9%) of the treated patients.

Concerning distribution of HLA class I alleles at study entry three remarkable effects were observed (Table 2): In HIV/HCV co-infected patients HLA-A*02 was less and HLA-B*51 more frequent than in the other patient groups and in the background population. The frequency of patients with HLA-B*57 was increased in patients with HIV infection (12.9%) and reduced in patients with hepatitis C (4.2%) as compared to patients with HIV/HCV co-infection (8%) and the healthy background population (9.2%). However, none of these differences was statistically significant after correction for multiple testing.

**Table 2:** Distribution of HLA-alleles at study entry.

**HLA-A locus n (%)**	**HIV(132)**	**HCV(120)**	**HIV/HCV(162)**	**Pearsons Chi-square**	**ReferencePopulation** §
HLA-A*01	32 (24.2)	32 (26.7)	39 (24.1)	0.865	35 (32.1)
HLA-A*02	77 (57.9)	57 (47.5)	70 (43.2)	0.039 *	54 (49.5)
HLA-A*03	26 (19.7)	33 (27.5)	45 (27.8)	0.219	33 (30.3)
HLA-A*11	13 (9.8)	18 (15.0)	14 (8.6)	0.214	11 (10.0)
HLA-A*24	20 (15.2)	28 (23.3)	37 (22.8)	0.178	18 (16.5)
HLA-A*32	11 (8.3)	10 (8.3)	18 (11.1)	0.640	5 (4.6)
HLA-A*68	12 (9.1)	13 (10.8)	18 (11.1)	0.837	10 (9.2)
**HLA-B locus n (%)**	**HIV(186)**	**HCV(120)**	**HIV/HCV (162)**	**Pearsons Chi-square**	
HLA-B*07	42 (22.6)	23 (19.2)	38 (23.5)	0.671	21 (19.2)
HLA-B*08	30 (16.1)	20 (16.7)	25 (15.4)	0.961	22 (20.1)
HLA-B*13	9 (4.8)	9 (7.5)	13 (8.0)	0.445	7 (6.4)
HLA-B*15	26 (14.0)	18 (15.0)	22 (13.6)	0.942	14 (12.8)
HLA-B*18	16 (8.6)	11 (9.2)	11 (6.8)	0.734	9 (8.2)
HLA-B*27	13 (7.0)	6 (5.0)	16 (9.9)	0.290	8 (7.3)
HLA-B*35	37 (19.9)	26 (21.7)	23 (14.2)	0.219	25 (22.9)
HLA-B*39	6 (3.2)	6 (5.0)	9 (5.6)	0.550	10 (9.2)
HLA-B*40	33 (17.7)	18 (15.0)	17 (10.5)	0.158	13 (11.9)
HLA-B*44	45 (24.2)	31 (25.8)	42 (25.9)	0.918	22 (20.2)
HLA-B*51	23 (12.4)	19 (15.8)	38 (23.5)	0.021 *	13 (11.9)
HLA-B*57	24 (12.9)	5 (4.2)	13 (8.0)	0.029 *	10 (9.2)

Analysis has been limited to alleles with > 5% prevalence in the study population

* p-values did not remain significant after correction for multiple testing [15].

^§^ DNA samples of 109 blood donors had been provided anonymously by the blood transfusion services and served to reflect the distribution of HLA alleles in the healthy Caucasian background population. Due to their anonymization follow-up data could not be obtained for the subjects in this reference group.

### Analysis of survival

Overall 60 patients had died during the observation period (HIV infection: n = 24 (12.9%), hepatitis C: n = 13 (10.8%), HIV/HCV co-infection: n = 23 (14.3%). Causes of death comprised infections (25%), malignancy (23.3%), cardiovascular events (18.3%), liver failure (15.0%), suicide or accident (6.7%), intoxication (3.3%) and renal failure (1.7%). In 4 patients (6.7%) the precise cause of death remained unclear. Infections (n = 8) and cardiovascular disease (n = 6) were the predominant causes of mortality in patients with HIV infection; hepatocellular carcinoma (n = 5) and end stage cirrhosis (n = 4; thereof 3 liver transplantations) in patients with chronic hepatitis C. In patients with HIV/HCV co-infection infectious complications (n = 5), advanced end stage cirrhosis (n = 5, thereof 2 liver transplantations), cardiovascular disease (n = 4) and liver cancer (n = 3) were the major causes of death. Of note, only 7 deaths due to AIDS-defining conditions were observed in patients with HIV and HIV/HCV infection (pneumocytis pneumonia n = 3, non-Hodgkin lymphoma n = 2, Castleman’s disease and cryptosporidiasis in 1 patient each).

HCV-infected patients revealed a trend for better survival under anti-HCV therapy (SVR: 7.0% mortality, treated patients without SVR: 13.6% mortality, untreated patients: 16.7% mortality, p = 0.051), while mortality in HIV infection was not related to any specific antiretroviral drug or class of drugs (data not shown).

**Fig 2:**
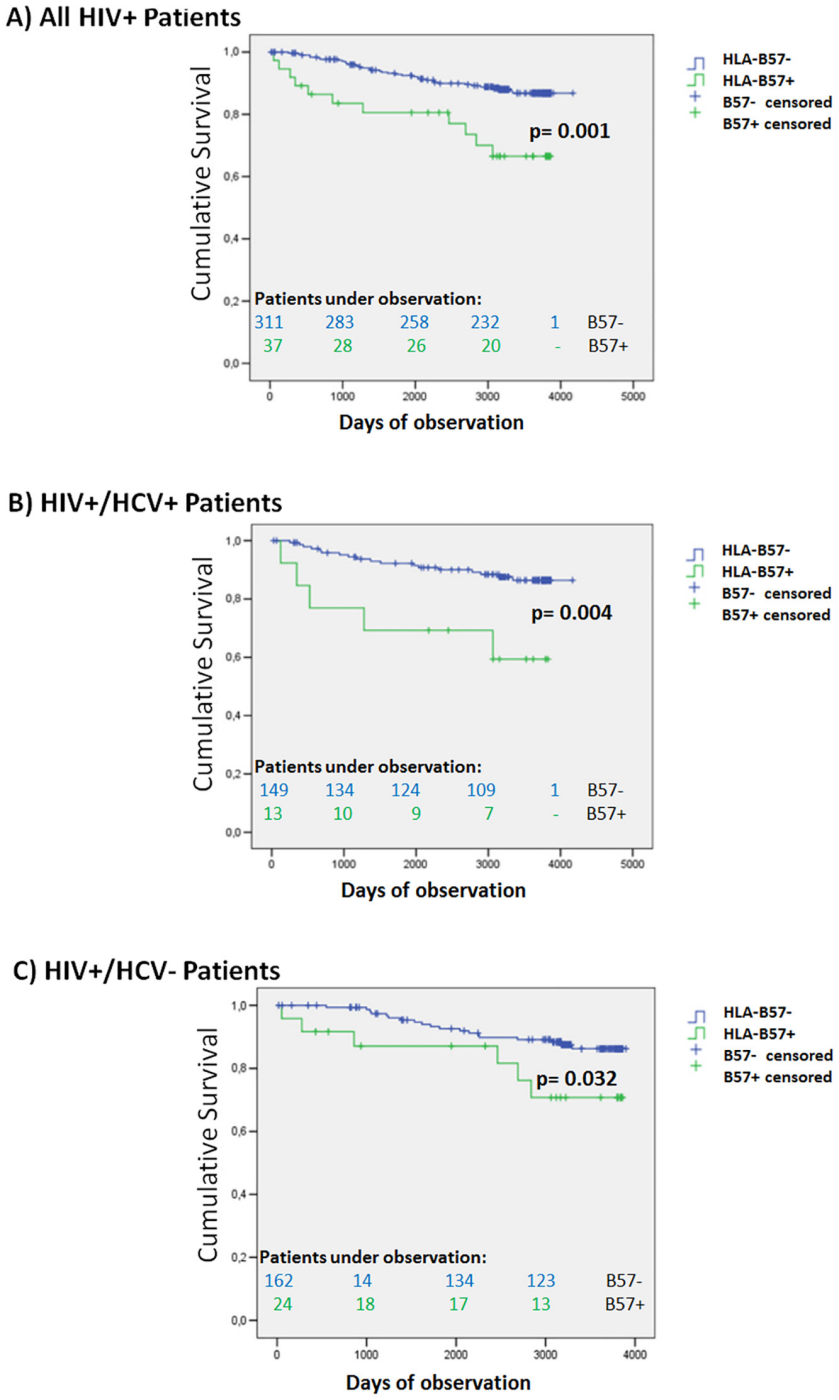
All-cause survival analysis stratified with respect to HLA-B*57. This figure shows Kaplan-Meier plots comparing all-cause-survival between HLA-B*57-positive (green lines) and HLA-B*57-negative patients (blue lines) in all HIV-infected patients (Fig 2A), and separately in the HIV-infected patients with (Fig 2B) and without HCV co-infection (Fig 2C). Vertical marks indicate censored patients. The Log-rank test was used to test statistical significances.

We also noted that 11/37 (29.7%) carriers of HLA-B*57 had died among the HIV and HIV/HCV infected patients, while all HLA-B*57 positive patients with hepatitis C had survived. Increased mortality of HLA-B*57-positive patients was confirmed by Kaplan Meier analysis in both HIV infection (p = 0.032) and HIV/HCV co-infection (p = 0.004) (Fig 2). Causes of death in the HLA-B*57-positive patients are listed in Table 3, illustrating that mortality was attributed to infectious complications in 8/11 patients, while infections accounted for only 5 of 24 deaths in the HLA-B*57 negative patients (p = 0.007). Of note, all HLA-B*57 positive patients except patient #3 (last HIV viral load 18594 copies/ml) had achieved HIV viral loads below the level of detection. Unlike HLA-B*57, we did not find significant differences in survival between positive and negative patients for any of the other studied HLA alleles (data not shown).

**Table 3:** Clinical features of the HLA-B*57-positive patients who had died.

Patient	Age	Diagnosis	Date of death	Cauof death	HAART	Hepatitis B	CD4 counts cells/μl before and after HAART
1	38	HIV	20.04.2005	unknown	emtricitabine, tenofovir, atazanavir, ritonavir^§^	-	before 417
							after 503
2	34	HIV	05.08.2005	candidiasis, pneumocystis pneumonia	lamivudine enfuvirtide tipranavir, ritonavir^§^	-	before 127
							after 134
3	57	HV #	21.09.2003	intestinal cryptosporidiosis	lamivudine, tenofovir, enfuvirtide, tipranavir, ritonavir^§^	-	before 116
							after 643
4	61	HIV	26.09.2009	sepsis *(MRSA)*	azidothymidine, lamivudine, atazanavir, ritonavir^§^	-	before 10
							after 42
5	57	HIV	20.05.2010	sepsis with intracranial bleeding	lamivudine, efavirenz, atazanavir, ritonavir^§^	-	before 238
							after 221
6*	41	HIV	18.08.2012	sepsis *(MRSA)*	tenofovir, emtricitabine, darunavir, ritonavir^§^	HBsAg-positive	before 227
							after 110
7	54	HIV/HCVgenotype 2b	12.08.2011	sepsis (*candida albicans*, *pseudomonas aeroginosa*)	didanosine, tenofovir, lopinavir, ritonavir^§^	-	before 70
							after 3
8	45	HIV/HCVgenotype1a	08.05.2004	hepatocellular cancer	didanosine, stavudine, efavirenz	HBsAg-positive	before 424
							after 1,108
9	44	HIV/HCVgenotype 3a	14.08.2006	sepsis (c*lostridium perfringens*)	stavudine, lamivudine, tenofovir, atazanavir, ritonavir^§^	-	before 152
							after 35
10	47	HIV/HCVgenotype 1b	19.06.2003	end stage liver cirrhosis	lamivudine, stavudine	-	before 923
							after 390
11	60	HIV/HCVgenotype unknown	19.09.2004	endocarditis, sepsis (*MRSA*)	darunavir, emtricitabine, tenofovir	-	before 702
							after 1,004

^§^ ritonavir booster dose

* This patient received a liver transplant in 2010. He died 2 years later from sepsis due to methicillin-resistant *staphylococcus aureus* (MRSA).

^#^ This patient had detectable HIV RNA (18.594 copies/ml) before death, all other patients had HIV RNA below the level of detection under HAART.

Multiple factors modify all cause-mortality. First, we checked if we could detect differences in clinical and viral parameters between HLA-B*57 positive and HLA-B*57 patients. CD4 counts at entry (HIV: 367 versus 474 cells/μl; HIV/HCV 377 versus 427 cells/μl) and at last follow-up (HIV: 492 versus 546 cells/μl; HIV/HCV 410 versus 446 cells/μl) tended to be lower in carriers of the HLA-B*57 allele. However, these differences were not statistically significant. We could not detect significant differences concerning age, gender, Fib-4 scores, platelets counts, HIV and HCV viral loads, liver enzymes and bilirubin (data not shown). HCV treatment rates were rather low, and HCV overall clearance rates did not differ between HLA-B*57- positive and negative patients (15.7% vs. 15.8%).

To further check if HLA-B*57 was an independent risk factor, we calculated separate conditional Cox logistic regression models for each patient group, summarized in Table 4. HLA-B*57 (p = 0.003), serum gamma-glutamyltranspeptidase (p = 0.030) and hepatic fibrosis as reflected by the Fib-4 score were independent risk factors in patients with HIV/HCV co-infection, while CD4 count, serum gamma-glutamyltranspeptidase and serum bilirubin—but not HLA-B*57—were independent predictors in HIV mono-infection. Serum AST and age were independent predictive parameters in chronic hepatitis C. Next, we performed logistic Cox regression analysis on all HIV-positive patients using “HCV infection” as an additional categorical covariate (Table 5), which confirmed HLA-B*57 (p = 0.002), gamma-glutamyltranspeptidase (p = 0.001), bilirubin (p = 0.007), and CD4 count (p = 0.037) as significant independent predictors of mortality. To exclude that treatment intensity had affected survival analysis, we re-calculated Kaplan-Meier analysis and Cox regression models including only HIV and HIV/HCV co-infected patients with HIV RNA below the detection limit. This re-analysis confirmed HLA-B*57 as a risk factor in HIV infection (log-rank p = 0.038) and HIV/HCV co-infection (log-rank and Cox regression p = 0.001 each; OR 3.663, 95% confidence interval 1.688–8.005).

**Table 4:** Independent risk factors for all-cause-mortality in HIV and HCV positive patients.

	Separate Cox regression models for each disease group:			
		OR	95%-CI	p-value
HIV-positive, HCV-negative patients	Billirubin	2.065	1.240–3.452	0.005
	CD4	0.998	0.996–1.000	0.022
	GGT	1.003	1.000–1.006	0.032
HIV/HCV-positive patients	HLA-B57	4.762	1.721–13.158	0.003
	Fib-4	1.118	1.023–1.223	0.014
	GGT	1.002	1.000–1.005	0.030
HCV-positive patients	Age	1.074	1.027–1.123	0.002
	AST	1.024	1.008–1.041	0.004

GGT = γamma-glutamyltranspeptidase

**Table 5:** Independent risk factors for all-cause-mortality in all HIV positive patients.

	Regression model involving all HIV positive patients in the cohort ^§^:			
		OR	95%-CI	p-value
HIV positive patients (all)	GGT	1.003	1.001–1.004	0.001
	HLA-B57	2.933	1.464–5.848	0.002
	Bilirubin	1.352	1.039–1.760	0.007
	CD4	0.999	0.997–1.000	0.037

^§^ HCV infection was an additional categorical covariate GGT = γamma-glutamyltranspeptidase

## Discussion

Taking into account the ethical and practical limitations for long-term survival studies in HIV-infected patients, our study design represents the best feasible strategy to analyze mortality in a real-life setting. Moreover, our study population revealed exceptionally good adherence and achieved HIV control below the level of detection in > 70% of HIV-infected patients.

Here, we report the novel and unexpected observation that in such a population HLA-B*57 may adversely affect long-term survival, particularly in HIV/HCV co-infection. The increased mortality risk associated with HLA-B*57 was identified by Kaplan-Meier analysis and confirmed by logistic regression analysis both in the patients with HIV/HCV co-infection and the entire population of HIV-infected patients. It is noteworthy that this increased risk was also confirmed, when our analysis was limited exclusively to HIV-infected patients with undetectable HIV replication, thus excluding insufficient treatment efficacy or poor adherence as potentially confounding factors. Our observation probably reflects the fact that the immunological advantage of HLA-B*57 seen in untreated HIV infection is lost when HIV replication falls below detectable levels, while residual HIV replication may still cause enhanced immune activation and a immune dysregulation syndrome [16–18]. In this context, immune stimulation by continued exposure to HCV antigens may particularly unmask unfavorable immunomodulation associated with HLA-B*57, so that this allele is then identified as an independent risk factor in multivariate statistical analysis. Of note, despite good control of HIV replication, recovery of CD4 counts was poor in eight of the 11 patients who had succumbed, suggesting that the effect associated with HLA-B*57 might be linked to impaired CD4 T cell recovery. In line with this assumption, CD4 counts were slightly lower in carriers of HLA-B*57.

Persistent immune activation [19], T cell exhaustion [20] and altered microbial translocation [21, 22] increase the risk for bacterial infections in HIV-infected patients with hepatitis C. In addition, increased mortality from non-viral infections and sepsis has been reported for HCV-infected patients also in immune-compromised conditions other than HIV infection, e.g. kidney transplantation [23], or liver transplantation [24]. However, the molecular and cellular mechanisms underlying the negative effect of HLA-B*57 in treated HIV and HIV/HCV infection remain unclear at present.

Although we cannot completely rule out that effects attributed to HLA-B57 in our analysis might be associated with genetic factors in linkage disequilibrium with the MHC such as HCP5 and ZNDR1, HLA alleles represent immune response genes that are sufficient to explain differential mortality. HLA-B*57 is a genetic host factor for spontaneous resolution of hepatitis C [12] and delayed disease progression in HIV infection [25], reflecting that presence HLA-B*57 probably leads to a more efficient / active immune system. Conversely, HLA-B*57 predisposes to immune over-activation syndromes, e.g. abacavir hypersensitivity (HLA-B*5701) [26] and spondylarthropathies (HLA-B*5703) [27].

Multiple factors determine long-time survival in HIV and HIV/HCV infection, which may be also potential sources of bias. Apart from using multivariate analysis we searched for major potentially confounding factors by several meticulous sub-analyses. However, we did not find evidence that differential survival between HLA-B*57 positive and negative patients was related to differences in disease severity, treatment intensity or risk behavior. We cannot fully exclude that selection before initiation of HAART might have introduced some survivor bias. In line with its established protective role, we found an increased frequency of HLA-B*57 positive patients with HIV infection at study entry, possibly reflecting better survival in the pre-HAART era, while this frequency was reduced in patients with chronic hepatitis C, possibly reflecting greater probability of spontaneous HCV elimination. However, these effects did not reach statistical significance after correcting for multiple comparisons. In addition, HLA-B*57 should have selected for patients with improved prognosis in HIV infection. Finally, the fact that the majority of our HIV/HCV-infected patients were hemophiliacs, who usually are less exposed to life-style related risk factors than other HIV-infected patient groups, further suggests that confounding effects are likely to have been small [28].

Further observations also seem to provide a hint for a varied heterogenous role of HLA-B*57 in HIV infection. Although the work of Brumme et al. did not claim an effect of HLA-B*57 on survival in HIV-infected patients receiving HAART [29], a closer look on their data revealed slightly worse survival in HLA-B*57 positive individuals. Their observations might miss statistical significance, because observation time was much shorter than in our study. In addition, they had not addressed HCV co-infection separately.

Next, Kuniholm et al. reported HLA-B*57 to be linked with reduced virological responses to HAART [30], and Rauch et al. reported impaired CD4 T cell recovery in HLA-B*57 positive patients [31], similar to our HLA-B57* positive patients.

In summary, our long-term observational data provide circumstantial evidence that HLA-B*57, a host genetic factor conferring immune-mediated protection in untreated HCV and HIV infection, may exert opposite effects on survival, when the disease course is altered by antiretroviral therapy. Thus, HLA-B*57 can exert ambiguous functions in infectious diseases depending on the precise environmental circumstances.

## Supporting Information

S1 File Original patient data for statistical analysis. (SAV)
